# Prenatal vitamin C and fish oil supplement use are associated with human milk microbiota composition in the Canadian CHILD Cohort Study

**DOI:** 10.1017/jns.2024.58

**Published:** 2024-09-26

**Authors:** Rana F. Chehab, Kelsey Fehr, Shirin Moossavi, Padmaja Subbarao, Theo J. Moraes, Piushkumar Mandhane, Russell J. de Souza, Stuart E. Turvey, Ehsan Khafipour, Meghan B. Azad, Michele R. Forman

**Affiliations:** 1 Department of Nutrition Science, Purdue University, West Lafayette, IN, USA; 2 Department of Pediatrics and Child Health, University of Manitoba, Winnipeg, MB, Canada; 3 Department of Physiology and Pharmacology, Snyder Institute for Chronic Diseases, University of Calgary, Calgary, AB, Canada; 4 Department of Pediatrics, Alberta Children’s Hospital Research Institute, University of Calgary, Calgary, AB, Canada; 5 International Microbiome Centre, University of Calgary, Calgary, AB, Canada; 6 Hospital for Sick Children, Department of Pediatrics & Physiology and Dalla Lana School of Public Health, University of Toronto, Toronto, ON, Canada; 7 Department of Pediatrics, Hospital for Sick Children and University of Toronto, Toronto, ON, Canada; 8 Department of Paediatrics, Faculty of Medicine and Dentistry, University of Alberta, Edmonton, AB, Canada; 9 Department of Health Research Methods, Evidence, and Impact, Faculty of Health Sciences, McMaster University, Hamilton, ON, Canada; 10 Population Health Research Institute, Hamilton Health Sciences Corporation, Hamilton, ON, Canada; 11 Department of Pediatrics, British Columbia Children’s Hospital and The University of British Columbia, Vancouver, BC, Canada; 12 Department of Animal Science, University of Manitoba, Winnipeg, MB, Canada; 13 Manitoba Interdisciplinary Lactation Centre (MILC), Children’s Hospital Research Institute of Manitoba, Winnipeg, MB, Canada; 14 Department of Food and Human Nutritional Sciences, University of Manitoba, Winnipeg, MB, Canada

**Keywords:** Breastmilk, CHILD Cohort Study, Diet, Microbiome, Pregnancy, Supplements

## Abstract

Maternal diet may modulate human milk microbiota, but the effects of nutritional supplements are unknown. We examined the associations of prenatal diet and supplement use with milk microbiota composition. Mothers reported prenatal diet intake and supplement use using self-administered food frequency and standardised questionnaires, respectively. The milk microbiota was profiled using 16S rRNA gene sequencing. Associations of prenatal diet quality, dietary patterns, and supplement use with milk microbiota diversity and taxonomic structure were examined using Wilcoxon signed-rank tests and multivariable models adjusting for relevant confounders. A subset of 645 mothers participating in the CHILD Cohort Study (originally known as the Canadian Healthy Infant Longitudinal Development Study) provided one milk sample between 2 and 6 months postpartum and used prenatal multivitamin supplements ≥4 times a week. After adjusting for confounders, vitamin C supplement use was positively associated with milk bacterial Shannon diversity (*β* = 0.18, 95% CI = 0.05, 0.31) and *Veillonella* and *Granulicatella* relative abundance (*β* = 0.54; 95% CI = 0.05, 1.03 and *β* = 0.44; 95% CI = 0.04, 0.84, respectively), and negatively associated with *Finegoldia* relative abundance (*β* = –0.31; 95% CI = –0.63, –0.01). Fish oil supplement use was positively associated with *Streptococcus* relative abundance (*β* = 0.26; 95% CI = 0.03, 0.50). Prenatal diet quality and dietary patterns were not associated with milk microbiota composition. Prenatal vitamin C and fish oil supplement use were associated with differences in the milk microbiota composition. Future studies are needed to confirm our findings and elucidate mechanisms linking maternal supplement use to milk microbiota and child health.

## Introduction

Human milk confers numerous benefits to the infant’s health including guiding the maturation of the immune system and modulating the gut microbiome development.^(1,2)^ It does so through its diverse constituents, including nutrients and bioactive components such as human milk oligosaccharides, lactoferrin, growth factors, and cytokines.^(3)^ In addition, the milk microbiome potentially impacts the infant microbiome development and guides the maturing immune system, adding to the list of pathways through which human milk can influence infant health.^(4,5)^

Numerous factors can shape the human milk microbiota composition, including maternal body mass index (BMI), delivery mode, lactation stage, mode and exclusivity of breast milk feeding, and peripartum antibiotic use.^(4,6,7)^ Although it is well established that maternal diet affects the human milk composition,^(8,9)^ especially the fatty acid profile,^(10)^ few studies investigated the effect of maternal diet on the milk microbial communities,^(11–14)^ and none have examined the effects of supplement use. Existing studies of maternal diet have been conducted among a small sample or among women with high rates of gestational glucose intolerance, while others did not adjust for potential confounding factors that influence the milk microbiota composition or utilised human milk samples prior to milk maturation.^(11–14)^

Pregnant and breastfeeding women are recommended to use multivitamin supplements since the nutrient needs of these women are generally greater than those of non-pregnant women, and the recommended intakes of some nutrients, such as folate and iron, are difficult to meet from food alone.^(15)^ Despite such recommendations and the widespread use of supplements during pregnancy,^(16)^ the effect of supplement use on the human milk microbiota composition is unknown.

To help fill this gap in the literature, we conducted a study among a diverse sample of 645 mothers participating in the CHILD Cohort Study in Canada who provided one milk sample between 2 and 6 months postpartum. The aim of this study is to examine the associations between prenatal diet quality, dietary patterns, and supplement use and the human milk microbiota composition. Findings on how prenatal maternal diet and supplement use are potentially associated with the human milk microbiome can help better inform nutrition guidelines of maternal intake during pregnancy.

## Subjects and methods

### Study design and subject selection

The CHILD Cohort Study (originally known as the Canadian Healthy Infant Longitudinal Development Study) is a longitudinal, prospective, population-based birth cohort study conducted across four regions in Canada: Vancouver, Edmonton, Manitoba, and Toronto.^(17)^ The main aim of the study is to examine the developmental origins of paediatric asthma and allergy. This study was approved by the Human Research Ethics Boards at McMaster University, the Hospital for Sick Children (Toronto) and the Universities of Manitoba, Alberta, British Columbia, and Purdue University.

**Fig. 1.**
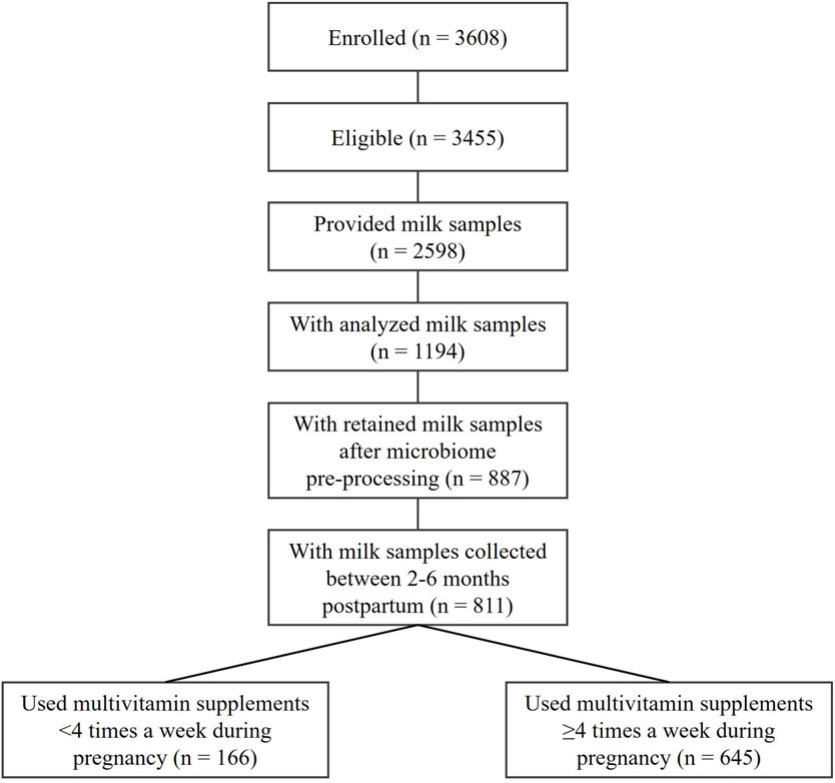
Flowchart for selecting mother-infant dyads in the CHILD Cohort Study included in the current analysis.

Women (*n* = 3608) in their second or third trimester of pregnancy were enrolled in the study between 2008 and 2012 (Fig. 1). Eligible subjects (*n* = 3455, 95.8%) had singleton pregnancies and delivered a healthy infant >35 weeks of gestation. A total of 2598 (75.2%) mothers provided human milk samples, 1194 (46.0%) of whom had their milk samples analysed for microbiota composition. Among those, 482 (40.4%) mothers were randomly selected from a representative subset of mother-infant dyads across the cohort,^(18)^ and the remaining 712 (59.6%) were from an additional subset enriched for maternal and infant health conditions (atopy, asthma, obesity).^(4)^ After microbiome pre-processing, the human milk samples of 887 (74.3%) mothers were retained in the analysis. Because lactation stage influences the human milk microbiota composition,^(19)^ we limited our analysis to the 811 (91.4%) mothers with milk samples collected between 2 and 6 months postpartum. Among the 811 mothers, 645 (79.5%) used multivitamin supplements ≥4 times a week throughout pregnancy. The 645 mothers constituted our primary sample of analysis because the chronic use of multivitamin supplements creates a relatively steady state in the body for each of the micronutrients in the multivitamin supplement and provided a more homogenous background to assess the role of additional individual micronutrient supplements on the microbiota.^(20)^

### Maternal and infant characteristics

Demographic characteristics of the mother including age, ethnicity, parity, and antibiotic use around the time of milk sample collection, and those of the infant including sex, delivery mode, gestational age at birth, and age at milk sample collection were documented from hospital records or from standardised questionnaires. Maternal pre-pregnancy BMI in kg/m^2^ was calculated using self-reported pre-pregnancy weight from standardised questionnaires and measured height abstracted from medical records.

Mothers completed standardised questionnaires about infant feeding practices at 3, 6, and 12 months postpartum. At the time of milk sample collection, breastfeeding status was classified as exclusive (human milk only) or partial (human milk supplemented with infant formula), and the mode of breast milk feeding was classified as all direct from the human (no feeding of pumped milk) or some pumped human milk (at least one serving of pumped human milk in the two weeks prior to milk sample collection).

### Prenatal diet quality, dietary patterns, and supplement use

Prenatal diet was assessed using a validated semi-quantitative food frequency questionnaire (FFQ) adapted from the Fred Hutchinson Cancer Center tool.^(21)^ The FFQ was self-administered and completed between 24 and 28 weeks of pregnancy. Details on the diet analysis were described elsewhere.^(22)^ Briefly, responses to the FFQ were linked to the United States Department of Agriculture nutrient composition database and modified for a Canadian setting^(23)^ to estimate total energy intake. Diet quality was assessed using the Healthy Eating Index 2010 (HEI-2010) score^(24)^ and dietary patterns were identified using principal component analysis (PCA) using the “psych” package (version 1.5.6) within R (version 3.1.2). Three dietary patterns emerged among the CHILD Cohort Study subjects: plant-based (characterised by dairy, legumes, vegetables, whole grains, and an aversion to meats), Western (characterised by fats, meats, processed foods, and starchy vegetables) and balanced (characterised by diverse sources of animal proteins [especially fish], vegetables, fruits, nuts and seeds).^(25)^ The PCA scores for each of the three patterns represented how close the mothers’ dietary intakes were to the dietary patterns. Positive PCA scores indicated adherence to the specific dietary pattern while negative scores indicated avoidance. Certain mothers had positive scores for more than one dietary pattern and were thus considered adherent to more than one pattern. Dietary pattern scores were adjusted to the mean energy intake of the cohort (2500 kcal/d) using the residual method.^(26)^ Women with implausible energy intakes (<500 or >6500 kcal/d) were excluded from the analysis of diet quality and dietary patterns.^(22)^

Mothers completed self-administered standardised questionnaires during pregnancy and after delivery about the frequency of use and dosage of supplements throughout pregnancy. Frequency of individual and multivitamin supplement use was reported based on the following options: never, 1–3 times a month, 1–3 times a week, 4–6 times a week, and every day. Dosage of prenatal supplements per day was reported for several but not all supplements and responses varied by questions for the individual supplements. Due to the unavailability of comparable responses on the frequency and dosage of supplement use, we categorised supplement use as never (reported never using the supplement during pregnancy) vs. ever (reported using the supplement at least once a month during pregnancy).

### Milk sample collection and microbiota profiling

Details on milk sample collection and analysis are described elsewhere.^(18)^ Briefly, mothers provided one milk sample which was a mix of fore- and hind milk from several feeds during a 24-hour period in a sterile milk container provided by the CHILD study. The milk samples were collected using hand expression or a pump and stored in the refrigerator at home for up to 24 hours. The study staff then transported and processed the milk samples and stored them at –80°C until analysis.

Milk microbiota was analysed at the University of Manitoba using 16S rRNA gene sequencing of the V4 hypervariable region on a MiSeq platform (Illumina, San Diego, CA, USA).^(18)^ Negative controls composed of sterile DNA-free water were used in sequencing library preparation, while positive controls consisted of DNA extracted from 8 species with known theoretical relative abundances (Zymo Research, USA, Cat# D6005).

Microbiota data pre-processing was previously described.^(27)^ Briefly, overlapping paired-end reads were processed with the DADA2 pipeline^(28)^ using the open-source software QIIME 2 v.2019.10 (https://qiime2.org).^(29)^ Unique amplicon sequence variants (ASVs) were assigned taxonomy and aligned to the 2019 release of SILVA v.138 SSURef NR99 at 99% sequence similarity.^(30)^ A three-step framework including (1) verification of sequencing accuracy, (2) contaminant removal and correction of batch variability, and (3) assessment of microbiome analysis repeatability was used for comprehensive quality control.^(27)^

Samples were rarefied to 8,000 sequencing reads per sample. ASVs with mean relative abundance ≤0.01% were discarded. ASVs were then agglomerated at the genus level and genera with mean relative abundance ≤0.1% and those present in ≤10% of the samples were discarded. ASVs and genera abundances were centred log-ratio transformed using the CoDaSeq package^(31)^ after zero imputation using a Bayesian-multiplicative replacement method.^(32)^ This dataset was used for downstream analysis unless otherwise stated.

### Statistical analysis

Frequency (%) and mean ± standard deviation (sd) were computed to describe the characteristics of the mother-infant dyads in this study. Differences between included vs. excluded mother-infant dyads and associations between maternal and infant characteristics and prenatal diet quality, dietary patterns, and supplement use were examined using *χ* ^2^ test. Diet quality was categorised as below vs. above the 50^th^ percentile (74.82) for HEI-2010 scores. Dietary patterns were categorised as adherence (positive PCA scores) vs. avoidance (negative PCA scores).^(25)^

Associations of prenatal diet quality, dietary patterns, and supplement use with human milk α diversity (Shannon index) and genera relative abundance were examined using Wilcoxon signed-rank tests and multivariable linear regression permutation models using lmPerm package v.2.1.0.^(33)^ Variables tested for their potential confounding effect included: parity, pre-pregnancy BMI, infant sex, delivery mode, and gestation age at birth as well as maternal antibiotic use, infant age, breastfeeding exclusivity, and mode of breast milk feeding at the time of milk sample collection. Based on the literature^(4,18,34)^ and the results of the univariable analysis, parity, pre-pregnancy BMI, and breastfeeding exclusivity and mode of breast milk feeding at the time of milk sample collection were adjusted for in the final multivariable models. The models also adjusted for the batches of milk samples, which were analysed separately.

The association with milk microbiota *β* diversity was examined using permutational analysis of variance (PERMANOVA) of a Bray-Curtis dissimilarity matrix derived using the ASV dataset via the *vegan* package (version 2.5.7).^(35)^ The Benjamini-Hochberg’s false discovery rate (FDR) was computed to correct the *P*-values for multiple comparisons of the genera relative abundance. A two-sided *P*-value <0.05 and a confidence interval (CI) not including 0 were considered statistically significant. Data analysis was conducted in R (version 4.0.3)^(36)^ using the *Phyloseq* package (version 1.36.0).^(37)^

## Results

### Maternal and infant characteristics, prenatal diet quality, dietary patterns, and supplement use

The 645 mothers included in this study had a mean ± sd age at delivery of 33.1 ± 4.2 years (range, 21.0–46.3 years); 80.0% were Caucasian, 58.0% were primiparous, 12.4% were obese, and 11.5% used antibiotics at the time of milk sample collection (Table 1). Fifty-four per cent of the infants were boys, 72.9% were born vaginally, 51.9% were exclusively breastfed, and 62.5% received some pumped milk at the time of milk sample collection. The mean ± sd age of the infants at the time of milk sample collection was 3.7 ± 0.8 months. As for maternal diet, 51.5% consumed a high-quality diet, 40.2% were adherent to a plant-based dietary pattern, 38.9% were adherent to a Western dietary pattern, and 43.7% were adherent to a balanced dietary pattern. In addition to using multivitamin supplements ≥4 times a week, mothers used the following supplements at least once a month: vitamin D (23.3%), vitamin C (5.0%), fish oil (20.6%), calcium (19.4%), folate (16.7%), and iron (12.1%).

**Table 1.** Characteristics of mother-infant dyads from the CHILD Cohort Study included in the current analysis

	Mother-infant dyads *N* = 645
Maternal characteristics	
Recruitment study centre, *n* (%)	
Edmonton	134 (20.8)
Toronto	174 (27.0)
Vancouver	171 (26.5)
Manitoba	166 (25.7)
Age at time of delivery (years)	
Mean ± SD	33.1 ± 4.2
Range	21.0 – 46.3
Ethnicity, *n* (%)	
Caucasian	516 (80.0)
Asian	98 (15.2)
Other	31 (4.8)
Primiparity, *n* (%)	374 (58.0)
Pre-pregnancy BMI, *n* (%)	
Normal weight (<25 kg/m^2^)	403 (62.5)
Overweight (25–29.99 kg/m^2^)	141 (21.9)
Obese (≥30 kg/m^2^)	80 (12.4)
Missing^a^	21 (3.2)
Antibiotic use at time of milk sample collection, *n* (%)	74 (11.5)
Infant characteristics	
Boys, *n* (%)	348 (54.0)
Vaginal delivery, *n* (%)	470 (72.9)
Term birth (≥39 weeks), *n* (%)	570 (88.4)
Exclusive breastfeeding at time of milk sample collection, *n* (%)	335 (51.9)
Some pumped breast milk feeding at time of milk sample collection, *n* (%)	403 (62.5)
Age at time of milk sample collection (months), mean ± SD	3.7 ± 0.8
Maternal prenatal diet	
High-quality diet^b^, *n* (%)	332 (51.5)
Adherence to plant-based pattern^c^, *n* (%)	259 (40.2)
Adherence to Western pattern^c^, *n* (%)	251 (38.9)
Adherence to balanced pattern^c^, *n* (%)	282 (43.7)
Maternal prenatal supplement ever use^d^	
Vitamin D, *n* (%)	150 (23.3)
Vitamin C, *n* (%)	32 (5.0)
Fish oil, *n* (%)	133 (20.6)
Calcium, *n* (%)	125 (19.4)
Folate, *n* (%)	108 (16.7)
Iron, *n* (%)	78 (12.1)

BMI, Body mass index; SD, standard deviation.

a Missing was analysed as a separate category in the multivariable models.

b High-quality diet: Healthy eating index-2010 scores ≥50^th^ percentile of 74.8.

c Adherence to a dietary pattern: Positive principal component analysis scores indicating that the mother’s dietary intake was similar to the components of the dietary pattern, which were as follows: plant-based (dairy, legumes, vegetables, whole grains, and an aversion to meats), Western (fats, meats, processed foods, and starchy vegetables) and balanced (diverse sources of animal proteins (especially fish), vegetables, fruits, nuts and seeds); categories are not mutually exclusive

d Supplement ever use: Use of the supplement at least once a month during pregnancy.

Mothers who consumed a high-quality diet and those who used prenatal vitamin D or fish oil supplements were less likely to have pre-pregnancy overweight or obesity (*P*-value < 0.001 for diet quality and *P*-value = 0.01 for vitamin D and fish oil supplement use). Furthermore, mothers who consumed a high-quality diet and those who used prenatal fish oil supplements were more likely to exclusively breastfeed at the time of milk sample collection (*P*-value = 0.02 for diet quality and *P*-value < 0.001 for fish oil supplement use). No other associations were observed between maternal or infant characteristics and prenatal diet quality, dietary patterns, and supplement use.

Few differences were noted in the characteristics of the mother-infant dyads included in our study compared to those who were excluded (*n* = 2963) (Supplementary Table 1). A larger proportion of the mothers included resided in Toronto (27.0% vs. 21.2%) and Vancouver (26.5% vs. 19.1%, *P*-value = 0.001), were Caucasian (80.0% vs. 70.0%, *P*-value = 0.005), and consumed a high-quality diet (51.5% vs. 34.8%, *P*-value <0.001). Further, more mothers included in the analysis used vitamin D supplements (23.3% vs. 13.9%, *P*-value = 0.001), fish oil supplements (20.6% vs. 10.4%, *P*-value <0.001), calcium supplements (19.4% vs. 11.6%, *P*-value = 0.003), and prenatal folate supplements (16.7% vs. 9.6%, *P*-value = 0.01) compared to those who were excluded.

### Human milk microbiota diversity and prenatal diet quality, dietary patterns, and supplement use

**Fig. 2.**
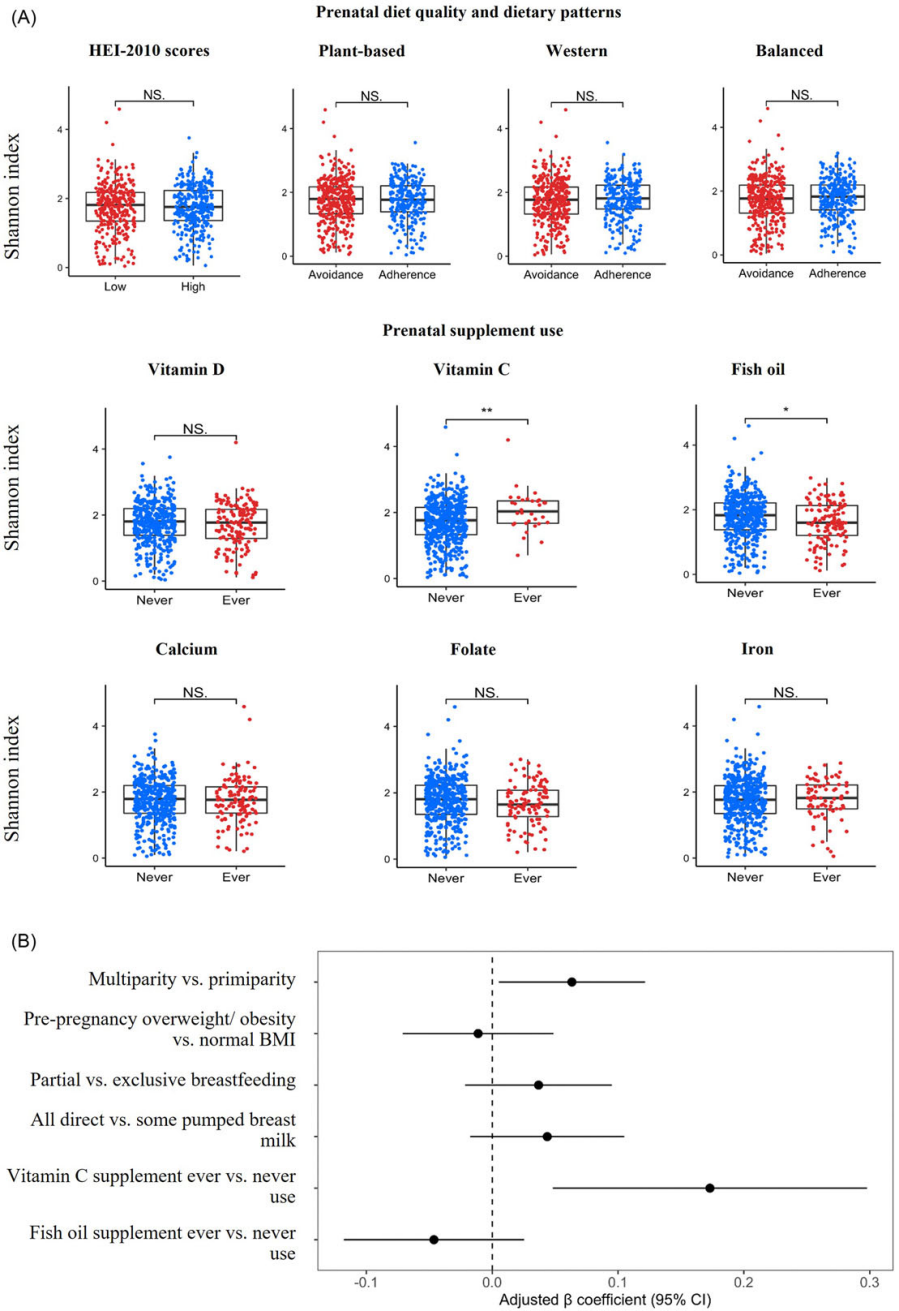
Human milk bacterial Shannon diversity by prenatal diet quality, dietary pattern, and supplement use among mothers in the CHILD Cohort Study. (a) Unadjusted associations examined using Wilcoxon-sign rank test. (b) Adjusted associations examined using multivariable linear regression permutation models adjusted for parity, pre-pregnancy BMI, breastfeeding exclusivity, mode of breast milk feeding at the time of milk sample collection, and fish oil and vitamin C supplement use; models were additionally adjusted for batch of analysis. Diet quality was examined using HEI-2010 scores categorised as low (<50^th^ percentile) vs. high (≥50^th^ percentile). Dietary patterns derived using PCA were examined as adherence (positive PCA scores) vs. avoidance (negative PCA scores). PCA scores reflected how closely the mother’s dietary intake was similar to the components of the dietary pattern, which were as follows: plant-based (dairy, legumes, vegetables, whole grains, and an aversion to meats), Western (fats, meats, processed foods, and starchy vegetables) and balanced (diverse sources of animal proteins (especially fish), vegetables, fruits, nuts and seeds). Supplement ever use was defined as use at least once a month during pregnancy. BMI, body mass index; CI, confidence interval; HEI, healthy eating index; PCA, principal component analysis. NS, not significant; *: *P*-value < 0.05; **: *P*-value < 0.01.

Mean ± sd of milk bacterial Shannon diversity was 1.74 ± 0.67. The milk bacterial Shannon diversity of mothers who used prenatal vitamin C supplements was higher (2.05 ± 0.61) than that of mothers who never used them (1.71 ± 0.65, *P*-value = 0.01) (Fig. 2a). The milk bacterial Shannon diversity of mothers who used prenatal fish oil supplements was lower (mean ± SD of milk bacterial Shannon diversity: 1.61 ± 0.64) than that of mothers who never used them (1.78 ± 0.68, *P*-value = 0.01). No significant differences in mean milk Shannon diversity were noted by diet quality or dietary patterns or use of other prenatal supplements.

After adjusting for relevant confounding factors, vitamin C supplement use was positively associated with milk bacterial Shannon diversity (*β* = 0.18; 95% CI = 0.05, 0.31) (Fig. 2b).

**Fig. 3.**
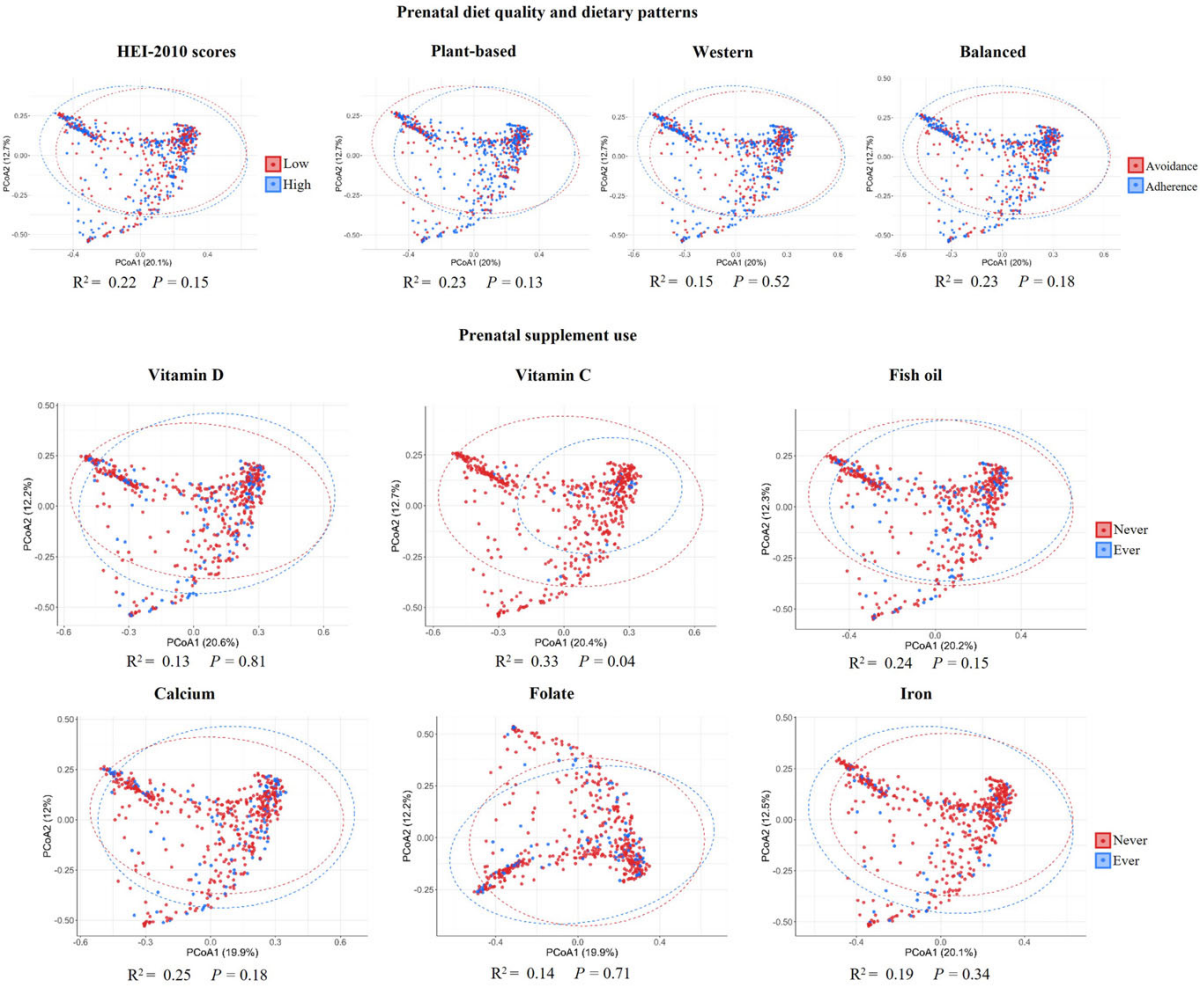
Human milk microbiota *β* diversity by prenatal diet quality, dietary patterns, and supplement use among mothers in the CHILD Cohort Study. *β* diversity assessed on Bray–Curtis dissimilarity matrix using permutational analysis of variance (PERMANOVA). Diet quality was examined using HEI-2010 scores categorised as low (<50^th^ percentile) vs. high (≥50^th^ percentile). Dietary patterns derived using PCA were examined as adherence (positive PCA scores) vs. avoidance (negative PCA scores). PCA scores reflected how closely the mother’s dietary intake was similar to the components of the dietary pattern, which were as follows: plant-based (dairy, legumes, vegetables, whole grains, and an aversion to meats), Western (fats, meats, processed foods, and starchy vegetables) and balanced (diverse sources of animal proteins (especially fish), vegetables, fruits, nuts and seeds). Supplement ever use was defined as use at least once a month during pregnancy. PCA, principal component analysis.

Significant, albeit small, differences were observed in milk microbiota *β* diversity for vitamin C supplement use (R^2^ = 0.33%, *P*-value = 0.04) (Fig. 3).

We conducted a sensitivity analysis among terms infants and mothers who did not use antibiotics. The results were materially unchanged: vitamin C supplement use was positively associated with milk bacterial Shannon diversity among term infants (*β* = 0.17; 95% CI = 0.04, 0.30) and women without antibiotic use (*β* = 0.19; 95% CI = 0.05, 0.32). In addition, vitamin C supplement use was associated with differences in milk microbiota *β* diversity among term infants (R^2^ = 0.344%, *P*-value = 0.05) and women without antibiotic use (R^2^ = 0.34%, *P*-value = 0.05).

### Taxonomic structure of human milk microbiota and prenatal diet quality, dietary patterns, and supplement use

Firmicutes were the predominant phylum in human milk in our study (mean relative abundance ± sd: 60.65% ± 35.17), followed by Proteobacteria (31.52% ± 37.39), Actinobacteria (5.99% ± 7.33), Bacteroidetes (1.57% ± 4.51) and Fusobaceria (0.13% ± 0.63). At the genus level, *Streptococcus* (mean relative abundance ± sd: 40.07% ± 31.39) and *Staphylococcus* (13.48% ± 21.59) were most abundant, followed by *Actinobacter* (10.11% ± 20.05) and *Pseudomonas* (7.45% ± 19.40). *Streptococcus, Staphylococcus,* and *Acinetobacter* were present in ≥90% of the 645 milk samples.

The relative abundance of *Streptococcus* was higher in the milk of mothers who used fish oil supplements compared to those who did not (mean relative abundance ± sd: 44.33% ± 32.24% vs. 38.59% ± 30.91%, *P*_FDR_ = 0.03). Similarly, the relative abundance of *Bifidobacterium* was higher in the milk of mothers who used fish oil supplements compared to those who did not (mean relative abundance ± sd: 0.52% ± 2.12% vs. 0.26% ± 1.33%, *P*_FDR_ = 0.03).

**Fig. 4.**
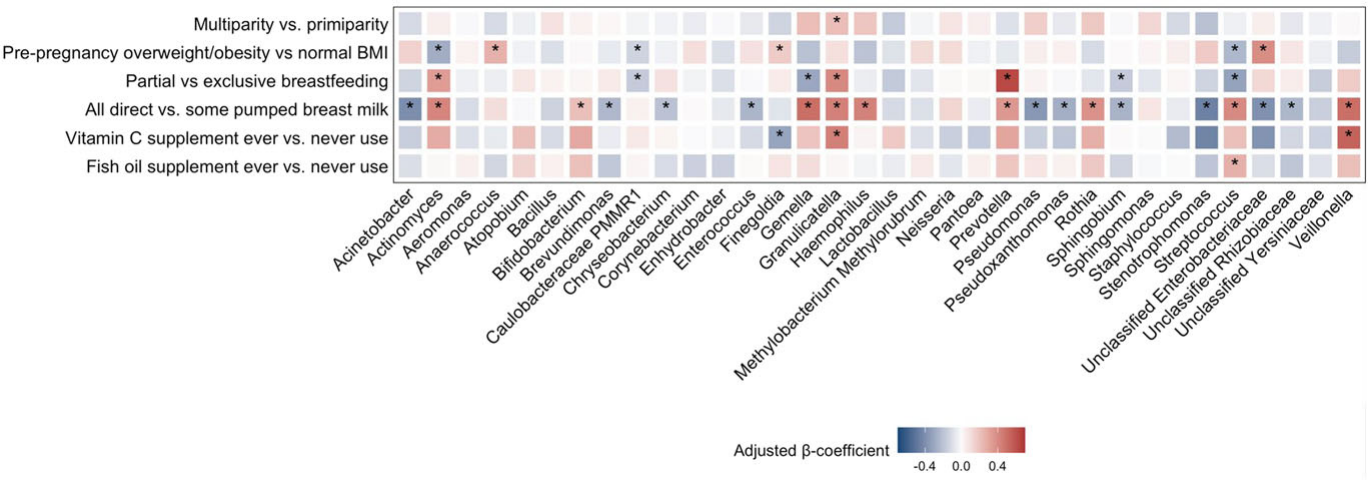
Genus relative abundance in the human milk of mothers in the CHILD Cohort Study by prenatal supplement use. Associations were examined using multivariable linear regression permutation models adjusted for parity, pre-pregnancy BMI, breastfeeding exclusivity, mode of breast milk feeding at the time of milk sample collection, and fish oil and vitamin C supplement use; models were additionally adjusted for batch of analysis. Supplement ever use was defined as use at least once a month during pregnancy. Genera relative abundance was centred log-ratio transformed. BMI, body mass index. *: Confidence interval not including 0.

After adjusting for relevant confounding factors, vitamin C supplement use was positively associated with *Veillonella* and *Granulicatella* relative abundance (*β* = 0.54; 95% CI = 0.05, 1.03 and *β* = 0.44; 95% CI = 0.04, 0.84, respectively), and negatively associated with *Finegoldia* relative abundance (*β* = –0.31; 95% CI = –0.63, –0.01) (Fig. 4). Fish oil supplement use was positively associated with *Streptococcus* relative abundance (*β* = 0.26; 95% CI = 0.03, 0.50).

## Discussion

In one of the largest studies of human milk microbiota, we report for the first time that prenatal vitamin C and fish oil supplement use were associated with milk microbiota composition, even after adjusting for confounding factors. Specifically, vitamin C supplement use was positively associated with milk bacterial Shannon diversity and with *Veillonella* and *Granulicatella* relative abundance and negatively associated with *Finegoldia* relative abundance. Fish oil supplement use on the other hand was positively associated with *Streptococcus* relative abundance. We limited our analysis to mothers who used multivitamin supplements ≥4 times a week since the chronic use of multivitamin supplements creates a relatively steady state in the body for each of the micronutrients in the multivitamin supplement.^(20)^

Despite the recommendations and widespread use of nutritional supplements during pregnancy,^(16)^ no previous studies have examined the associations between supplement use and the human milk microbiota composition. The few studies on maternal diet and the milk microbiome, although they do not account for prenatal supplement use, provide some insight into the associations between vitamin C and fish oil and the milk microbiota composition.^(13,14)^ The vitamin C composition of the prenatal diet of 94 healthy women in Brazil was associated with differences in milk microbiota *β* diversity,^(13)^ similar to our study. Further, vitamin C intake in the diet during pregnancy was positively correlated with *Staphylococcus* presence,^(13)^ while in our study, vitamin C supplement use was positively associated with *Veillonella and Granulicatella* and negatively associated with *Finegoldia.* It is worth noting that the sample size of mothers using vitamin C supplements in our study is small (5%); yet the consistent association of vitamin C in prenatal supplements and diet with the human milk microbiota composition warrants further investigation. In the MAternal MIcrobes (MAMI) study among 120 healthy mothers in the Spanish Mediterranean area, prenatal dietary omega-3 polyunsaturated fatty acids, including eicosapentaenoic acid, docosapentaenoic acid, and docosahexaenoic acid are major fatty acids in fish oil, were positively associated with *Streptococcus* and *Gemella* abundance, while total polyunsaturated fatty acids were negatively associated with *Bifidobacterium* abundance.^(14)^ We found positive associations between fish oil supplement use and *Staphylococcus* and *Bifidobacterium* abundance*. Bifidobacterium* is the hallmark of breastfed infant gut microbiota, suggesting a possible link between human milk and the neonatal gut;^(38)^ however, the association with *Bifidobacterium* abundance was not significant after adjusting for relevant confounders. More studies are needed to reproduce our findings, to examine the combined effects of these nutrients from diet and supplements during pregnancy and lactation on human milk nutrient and microbiota composition, and to elucidate the functional significance of the observed associations in relation to maternal and infant health.

We did not find significant associations between prenatal diet quality and dietary patterns (plant-based, Western, and balanced) and the human milk microbiota composition. Few studies examined the association of maternal diet with the milk microbiota composition.^(11–14)^ In the study among Brazilian women, stronger associations were noted between the milk microbiota composition and the nutrient composition of the diet during pregnancy compared to that during lactation, supporting our analysis of maternal diet and supplement use during pregnancy.^(13)^ The MAMI study examined prenatal diet in terms of diet clusters (Cluster I characterised by plant protein, fibre, and carbohydrates and Cluster II characterised by animal protein and lipids) rather than individual nutrients and found that the diet clusters were associated with the milk microbiota composition more than other factors including breastfeeding exclusivity and delivery mode.^(14)^ Cluster I was associated with higher bacterial Shannon diversity and higher relative abundance of *Staphylococcus, Lactobacillus,* and *Bifidobacterium* and lower abundance of *Bacteroides* and *Escherichia/Shigella* compared to Cluster II.^(14)^ The nutrient composition of Cluster I seems to be most similar to the plant-based dietary pattern derived among the CHILD Cohort Study subjects; however, we did not detect any associations between this pattern and milk microbiota composition. Future studies of the associations between prenatal dietary patterns and the human milk microbiota composition are needed.^(39)^

### Study strengths and limitations

Our study has several strengths. The CHILD Cohort Study is conducted among a diverse sample of mother-infant dyads and is among the largest studies with available human milk microbiota data to date. The longitudinal prospective cohort study design provided the opportunity to examine prenatal maternal diet and supplement use prior to milk sample collection. Some limitations are worth noting. This is an observational study among a Canadian cohort of women whose place of residence might have influenced both maternal diet and supplement use and the milk microbiota, thus the findings may not be generalisable to other settings. However, the characteristics of the women included in our analytic sample were similar to those in the overall CHILD cohort, and the majority of the women consumed a healthy diet and used multivitamin supplements prenatally, which enhances comparability of our findings to other similar cohorts. We did not account for the dose, frequency, and form of the prenatal supplements nor did we account for postnatal supplement use. Nevertheless, previous studies that examined prenatal and postnatal diet noted stronger associations between prenatal diet and the milk microbiota composition.^(13)^ Our dietary patterns were also not mutually exclusive—i.e. one participant received scores for all three patterns; and only the patterns for which a person “positively” adhered to were ascribed to them. This enhanced interpretation and accommodated the fact that dietary patterns are not “all or nothing.” Future studies are ongoing to explore alternative approaches to dietary pattern analyses in the CHILD cohort. Finally, we did not examine how dietary sources of vitamin C and fish oil were associated with the human milk microbiota composition. Future studies are needed to better understand the combined effect of maternal nutrient intake from diet and supplements on the human milk microbiota composition. Despite the current limitations, our exploratory study begins to fill a gap in the understanding of the associations between maternal dietary patterns and supplement use and the human milk microbiota.

In conclusion, fish oil and vitamin C supplement use were associated with the human milk microbiota composition among mothers in the CHILD Cohort Study who used multivitamin supplements ≥4 times a week during pregnancy. Such findings can inform future hypotheses and intervention trials to elucidate mechanisms linking maternal diet and supplement use to the human milk microbiota. A better understanding of these mechanisms and their effects on child health will help inform recommendations on maternal intake during pregnancy. Further research is warranted to test whether our findings are reproducible in other populations and to examine the effects of frequency and dose of supplement use on the associations with the human milk microbiota and child health. Future research is also needed to examine the associations with other human milk components and the infant gut microbiota and overall health, as well as to examine the associations beyond the community profile to explore function and metabolites produced by the microbes.

## Supporting information

Chehab et al. supplementary material Chehab et al. supplementary material

## Data Availability

A list of variables available in the CHILD Cohort Study is available at https://childstudy.ca/for-researchers/study-data/. Researchers interested in collaborating on a project and accessing CHILD Cohort Study data should contact the Study’s National Coordinating Centre (NCC) to discuss their needs before initiating a formal request. To contact the NCC, please email child@mcmaster.ca. More information about data access for the CHILD Cohort Study can be found at https://childstudy.ca/forresearchers/data-access/.

## Abbreviations

**ASVs**, amplicon sequence variants; **CI**, confidence interval; **FDR**, false discovery rate; **FFQ**, food frequency questionnaire; **HEI-2010**, Healthy Eating Index 2010; **PCA**, principal component analysis; **PERMANOVA**, permutational analysis of variance; **sd**, standard deviation.

## Supplementary material

The supplementary material for this article can be found at https://doi.org/10.1017/jns.2024.58
