# Supplementary material for: Prenatal vitamin C and fish oil supplement use are associated with human milk microbiota composition in the Canadian CHILD Cohort Study
Source: J Nutr Sci. 2024 Sep 26;13:e53. doi: 10.1017/jns.2024.58 (PMC11428054; doi:10.1017/jns.2024.58)
Supplement: Chehab et al. supplementary material [file S2048679024000582sup001.docx]

**Online Supplemental Material**

**Supplementary Table 1.** Comparison of characteristics of included versus excluded mother-infant dyads

|  | **Included dyads**  **N=645** | **Excluded dyads**  **N=2963** | ***P-*value** |
| --- | --- | --- | --- |
| **Maternal characteristics** |  |  |  |
| Recruitment study center, n (%) |  |  |  |
| Edmonton | 134 (20.8) | 655 (22.1) | 0.001 |
| Toronto | 174 (27.0) | 628 (21.2) |  |
| Vancouver | 171 (26.5) | 567 (19.1) |  |
| Manitoba | 166 (25.7) | 855 (28.9) |  |
| Age at time of delivery (years), mean ±SD | 33.1 ± 4.2 | 32.2 ± 4.8 | 0.07 |
| Ethnicity, n (%) |  |  |  |
| Caucasian | 516 (80.0) | 2074 (70.0) | 0.005 |
| Asian | 98 (15.2) | 345 (11.6) |  |
| Other | 31 (4.8) | 221 (7.5) |  |
| Primiparity, n (%) | 374 (58.0) | 1386 (46.8) | 0.05 |
| Pre-pregnancy BMI, n (%) |  |  |  |
| Normal weight (<25 kg/m^2^) | 403 (62.5) | 1459 (49.2) | 0.39 |
| Overweight (25-29.99 kg/m^2^) | 141 (21.9) | 493 (16.6) |  |
| Obese (≥30 kg/m^2^) | 80 (12.4) | 360 (12.1) |  |
| **Infant characteristics** |  |  |  |
| Boys, n (%) | 348 (54.0) | 1386 (46.8) | 0.51 |
| Vaginal delivery, n (%) | 470 (72.9) | 1951 (65.8) | 0.30 |
| **Maternal prenatal diet** |  |  |  |
| High-quality diet^1^, n (%) | 332 (51.5) | 1031 (34.8) | <0.001 |
| Adherence to plant-based pattern^2^, n (%) | 259 (40.2) | 941 (31.8) | 0.60 |
| Adherence to Western pattern^2^, n (%) | 251 (38.9) | 1004 (33.9) | 0.24 |
| Adherence to balanced pattern^2^, n (%) | 282 (43.7) | 1059 (35.7) | 1.00 |
| **Maternal prenatal supplement ever use^3^** |  |  |  |
| Vitamin D, n (%) | 150 (23.3) | 413 (13.9) | 0.001 |
| Vitamin C, n (%) | 32 (5.0) | 106 (3.6) | 0.78 |
| Fish oil, n (%) | 133 (20.6) | 309 (10.4) | <0.001 |
| Calcium, n (%) | 125 (19.4) | 345 (11.6) | 0.003 |
| Folate, n (%) | 108 (16.7) | 284 (9.6) | 0.01 |
| Iron, n (%) | 78 (12.1) | 280 (9.4) | 0.94 |

BMI: body mass index; SD: standard deviation.

*P*-value calculated using χ square test for categorical variables and independent sample t test for continuous variables.

^1^High-qiality diet: Healthy eating index-2010 scores ≥50^th^ percentile of 74.8.

^2^Adherence to a dietary pattern: Positive principal component analysis scores indicating that the mother’s dietary intake was similar to the components of the dietary pattern, which were as follows: plant-based (dairy, legumes, vegetables, whole grains, and an aversion to meats), Western (fats, meats, processed foods, and starchy vegetables) and balanced (diverse sources of animal proteins (especially fish), vegetables, fruits, nuts and seeds).

^3^Supplement ever use: Use of the supplement at least once a month during pregnancy.
